# Anti-Inflammatory and Anti-Oxidative Synergistic Effect of Vitamin D and Nutritional Complex on Retinal Pigment Epithelial and Endothelial Cell Lines against Age-Related Macular Degeneration

**DOI:** 10.3390/nu13051423

**Published:** 2021-04-23

**Authors:** Maria Hernandez, Sergio Recalde, Jorge González-Zamora, Valentina Bilbao-Malavé, Manuel Sáenz de Viteri, Jaione Bezunartea, Maite Moreno-Orduña, Idoia Belza, Jesús Barrio-Barrio, Patricia Fernandez-Robredo, Alfredo García-Layana

**Affiliations:** 1 Retinal Pathologies and New Therapies Group, Experimental Ophthalmology Laboratory, Department of Ophthalmology, Clinica Universidad de Navarra, 31008 Pamplona, Spain; srecalde@unav.es (S.R.); jgzamora@unav.es (J.G.-Z.); vbilbao@unav.es (V.B.-M.); msaenzdevit@unav.es (M.S.d.V.); jbezunartea@unav.es (J.B.); maimoreno@unav.es (M.M.-O.); idoiabelza@unav.es (I.B.); jbarrio@unav.es (J.B.-B.); pfrobredo@unav.es (P.F.-R.); aglayana@unav.es (A.G.-L.); 2 Navarra Institute for Health Research, IdiSNA, 31008 Pamplona, Spain; 3 Red Temática de Investigación Cooperativa Sanitaria en Enfermedades Oculares (Oftared), 31008 Pamplona, Spain

**Keywords:** vitamin D, nutritional complex, AMD, inflammation, oxidative stress, retina

## Abstract

Age-related macular degeneration (AMD) is a multifactorial disease of the retina featured by dysfunction of retinal pigmented epithelial (RPE) and loss of photoreceptor cells under oxidative stress and inflammatory conditions. Vitamin D and antioxidants have beneficial effects against retinal degenerative diseases, such as AMD. We investigated the impact of associating vitamin D (ND) with a nutritional antioxidant complex (Nutrof Total^®^; N) on oxidative stress and inflammation-like induced conditions by H_2_O_2_ and LPS, respectively, in human retinal epithelial (ARPE-19) and human retinal endothelial (HREC) cells. Application of either N or ND treatments to H_2_O_2_-induced media in ARPE-19 cells counteracted late apoptosis, attenuated oxidative DNA damage, and increased cell proliferation. Significant reduction in the expression levels of MCP1, IL-8, and IL6 cytokines was observed following application of either N or ND treatments under LPS-induced conditions in ARPE-19 cells and in MCP-1 and IL12p70 cytokine levels in HREC cells. ND and not N revealed significant downregulation of IFNγ in ARPE-19 cells, and of IL-6 and IL-18 in HREC cells. In conclusion, adding vitamin D to Nutrof Total^®^ protects in a synergistic way against oxidative and inflammatory stress-induced conditions in retinal epithelial and endothelial cells.

## 1. Introduction

Age-related macular degeneration (AMD) is a chronic progressive degenerative eye disease that gradually destroys the macula, leading to irreversible blurred vision or vision loss [1,2,3]. AMD is the primary cause of visual impairment in the elderly, and without an effective treatment, the prevalence of AMD is estimated to increase by 40% by 2040 [4].

AMD is clinically classified as early-, intermediate-, or late-stage depending on the size of drusen and on pigmentary abnormalities. Late-stage is linked to neovascular or geographic atrophy AMD [3,5]. Neovascularization of the retina is implicated in the development of abnormal blood vessels that penetrate Bruch’s membrane from the choroid and lead to progressive damage of the retinal pigmented epithelium (RPE) cell layer and of the photoreceptors in proximity [2]. The pathogenesis of AMD is well described as the outcome of complex multifactorial interactions involving genetic, metabolic, functional, and environmental factors [6,7,8,9,10,11,12,13]. These factors result in overproduction of reactive oxygen species, and thus accumulation of oxygen radicals and oxidative stress in RPE [14,15] can lead to dysfunction of RPE, which subsequently affects the nutrition of photoreceptors, resulting in angiogenesis and choroidal neovascularization (CNV) [16,17].

Currently, anti-vascular endothelial factor (VEGF) therapy is administered as an intravitreal injection to regress development to wet AMD [1], while no preventive therapy exists for dry AMD. Treatment with anti-VEGF agents is not always effective, with certain patients experiencing recurrent symptoms of exudation and additional injections leading to patient compliance issues, undesirable side effects, and increased financial burden to the healthcare system or the patient [18].

Therefore, antioxidant formulations (consisting primarily of vitamins C and E) and mineral supplementation (zinc, copper) have been examined in two earlier sequential clinical trial studies (Age-Related Eye Disease Study (AREDS and AREDS2)), sponsored by the National Eye Institute, showing reduced 5-year risk on progressing from intermediate to advanced AMD by about 25% in participants taking antioxidant formulation along with zinc and copper compared to those originally assigned to placebo [19,20]. The nutritional antioxidant complex in this study contains antioxidant vitamins and trace elements, natural components (lutein and zeaxanthin), and resveratrol, and is enriched in fish oil (Table S1).

Previous studies have demonstrated a relationship between deficient levels of vitamin D and onset or development of AMD [16,21,22,23,24,25,26,27,28,29,30], suggesting that vitamin D administration can be protective or preventive from progression to neovascular AMD. Recent studies have reported expression of vitamin D receptors throughout the eye, including the retina [31,32], where they are expressed by human retinal pigment epithelial cells (ARPE-19) [31,33] and retinal endothelial cells [34], suggesting vitamin D’s functional significance in the ocular system [35]. Moreover, a recent study has reported expression of vitamin D-synthesizing components in ARPE-19 and HREC cells [36]. The expression of vitamin D receptors in RPE cells has been previously demonstrated by our group, using PCR experiments [36], and by other investigators who demonstrated by immunohistochemical staining presence of vitamin D receptors throughout the RPE [37] as well as by whole-transcriptome expression profiles in the RPE–choroid region collected from human donors [38]**.**

Numerous observational studies have investigated the association between vitamin D and AMD, with current data remaining inconclusive and controversial. For example, a meta-analysis of 11 observational studies demonstrated an inverse relationship between low levels of plasma vitamin D levels and late AMD [23], while others with early AMD [21,39]. However, a recent review reported no evidence of a relationship between vitamin D levels and risk for AMD [40,41,42,43]. Additionally, an inconsistent relationship exists between vitamin D status and AMD in the epidemiological context to date. For example, in a prospective 18-year follow-up analysis of participants in the Atherosclerosis Risk in Communities Study, a statistically inversed relationship was found to exist between incidence of AMD and low vitamin D concentrations [26]. A protective relationship between early AMD and vitamin D serum levels was observed in a cohort of African Americans and Caucasians [27] and in non-Hispanic white, non-Hispanic black, and Mexican Americans. However, it was only statistically significant among non-Hispanic whites [21]. On the other hand, no overall statistically significant relationship was described in a survey of Korean adults [43] in a retrospective cohort of Medicare beneficiaries [41] and in an elderly cohort of French participants [42] between vitamin D concentrations and early or late AMD.

In this study, we would like to provide additional evidence on the potential impact of vitamin D in AMD. Therefore, vitamin D was added to the nutritional antioxidant complex to investigate both their potential synergistic effect and individual outcome on oxidation and inflammation-induced damage, which play a key role in retinal degenerative diseases including AMD. To this end, we examined the potential protective effect of these treatments on cell integrity, cell proliferation, DNA oxidative damage, apoptosis, and cytokine expression levels in ARPE-19 and human retinal endothelial cells (HREC).

## 2. Materials and Methods

### 2.1. Cell Culture

The same cell lines and culture system were used as previously described [36]. Briefly, human retinal pigment epithelial cells, ARPE-19 (CRL-2302, ATCC, Manassas, VG, USA; 3 passages) were grown to confluence in a standard incubator in DMEM (D6429, Sigma-Aldrich, St Louis, MO, USA) containing 10% fetal bovine serum (FBS; 10270106 Gibco^TM^ ThermoFisher, Paisley, UK), 1% fungizone (Gibco^TM^), and penicillin–streptomycin (Gibco^TM^). Human retinal endothelial cells (HREC; p10880, Innoprot, Vizcaya, Spain) after being seeded in T75 flasks were covered with 1 mg/mL of fibronectin (Innoprot, p8248) and grown to confluence in a standard incubator in Endothelial Cell Medium (Innoprot) containing 5% FBS (Innoprot), 1% Endothelial Cell Grow Supplement (ECGS; Innoprot), and penicillin–streptomycin solution (Innoprot).

### 2.2. Stable Cell Line Phenotypic Characterization

As previously described [36], immunofluorescence with RPE65 (1:100, 78036, Abcam, Cambridge, USA) and caveolin (1:250, 3238S, Cell Signalling, Danvers, MA, USA) was performed for ARPE-19 and HREC cells, respectively. Briefly, 100,000 ARPE-19 and 50,000 HREC cells were seeded on a 10 mm dish (Menzel-Glaser, Waltham, MA, USA) and following cellular fixation with cold methanol, cells were blocked with 1% BSA, 0.5% Triton X-100, 0.2% sodium azide, and 1% FBS for 1 h at 4 °C. Cells were then incubated with the primary antibodies at 4 °C for 24 h and subsequently incubated for 1 h at room temperature with the secondary fluorescent antibodies goat anti-mouse 488 (1:250, A11029, Life Technologies, Gaithersburg, MD, USA) for caveolin marker and donkey anti-rabbit 488 (1:250, A21206, Invitrogen, Carlsbad, CA, USA) for RPE65 marker, accordingly. Nuclei were labelled with DAPI (6-diamidino-2-phenylindole; Sigma-Aldrich, St Louis, MO, USA). Fluorescent images were obtained using a confocal microscope (LSM800, Zeiss, Oberkochen, Germany).

### 2.3. Experimental Design

#### 2.3.1. Oxidative Stress and Inflammatory-Like Conditions

ARPE-19 and HREC cell lines were incubated for 2 h with hydrogen peroxide (H_2_O_2_; either 600, 1000, or 1600 µM, according to the experiment; Panreac, Barcelona, Spain) to induce in vitro oxidative stress, and for 24 h with lipopolysaccharide (LPS; 20 µg/mL for ARPE-19 and 50 µg/mL for HREC cells; Sigma-Aldrich, St. Louis, MO, USA) to induce an inflammatory response, respectively. Saline was used as negative control. Cells were collected at the end of the induction time.

#### 2.3.2. Treatments with the Nutritional Antioxidant Complex (Nutrof Total^®^) and Vitamin D

During the last hour of the induction time, prior to cell harvesting, cells were treated with either Nutrof Total^®^ (N; 62.34 μg/mL, see Table S1 for composition; from Thea Laboratoires, Clermont-Ferrand, France) or Nutrof Total^®^ plus 1 nM vitamin D (ND) at a total equivalent concentration of 62.34 μg/mL. This concentration was used for both N and ND treatments in our experiments in order to have consistency in our comparisons between N and ND treatments.

### 2.4. Cell Viability/Cytotoxicity Assay (MTT), and Proliferation Assay (Bromodeoxyuridine, BrdU)

Cell viability/cytotoxicity and cell proliferation in ARPE-19 and HREC cell lines was determined by the 3-(4,5-dimethylthiazol-2-yl)-2,5-diphenyltetrazolium bromide (MTT) reduction assay CellTiter 96 Aqueous One Solution Cell Proliferation Assay (Promega, Madison, WI, USA), and by the Calbiochem BrdU Cell Proliferation Assay (Calbiochem, La Jolla, CA, USA), respectively, according to the manufacturer’s instructions, as previously described [36].

Cells were exposed to N and ND treatments for 24 h to examine their effect on cytotoxicity. Two different concentrations for vitamin D (1 nM and 5 nM) were selected as being safe [36]. Consequently, the equivalent ND concentrations were calculated on the basis of these vitamin D values (ND1: 62.34 µg/mL and ND5: 311.7 µg/mL). To examine cell proliferation, cell lines were exposed to 1000μM H_2_O_2_ for 2 h to induce oxidative stress conditions, while N and ND treatments (62.34 μg/mL each) were applied individually to H_2_O_2_-induced media during the last hour of the induction time.

### 2.5. Zonula Occludens (ZO-1) Immunofluorescence for Cell Structure and Integrity

Immunofluorescence against zonula occludens-1 (ZO-1) was performed to assess the effect of both N and ND treatments on intercellular tight junctions. ARPE-19 cells were seeded at a density of 100,000 cells per well on laminin-coated polycarbonate membrane cell culture inserts (Corning Life Science, Tewksbury, MA, USA) and were grown in 1% serum-free DMEM for 4 weeks. Immunofluorescence was then performed using a ZO-1 anti-rabbit Alexa Fluor 594 antibody (1:100, 339194, Invitrogen- Life Technologies, Carlsbad, CA, USA) diluted in blocking buffer following the same protocol as previously described [36]. DAPI (6-diamidino-2-phenylindole; Sigma-Aldrich) was used to stain cell nuclei. Images were obtained with a laser scanning confocal imaging system (LSM800, Zeiss, Oberkochen, Germany). N and ND treatments (62.34 µg/mL) were added individually to the cell line in order to be compared to the saline group, and were also added with either H_2_O_2_ (1600 µM) or LPS (as described in Section 2.3) in order to identify potential recovery effects on cell integrity.

### 2.6. Cell Apoptosis Assay

Apoptosis assay was performed as previously described [36]. Briefly, apoptosis in ARPE-19 cells was detected in cultured plates using in situ cell death detection kit with TMR Red (#12 156792910, Roche, West Sussex, UK) following the manufacturer’s instructions. Nuclei were labelled with DAPI and images were obtained using a confocal microscope (LSM800, Zeiss, Oberkochen, Germany). N and ND treatments (62.34 µg/mL) were added individually to ARPE-19 cells in order to be compared to the saline group, and were also added with either H_2_O_2_ (600 µM) or LPS (as described in Section 2.3) in order to identify potential preventive effects on cell apoptosis.

### 2.7. Evaluation of Oxidative DNA Damage by 8-Hydroxidioguanosine (8-OHdG)

Oxidative damage to DNA was measured under oxidative stress conditions in ARPE-19 supernatants subjected to H_2_O_2_ (1000 µM), as described in Section 2.3. During the last hour of the induction time, cells were exposed to either N or ND treatments (62.34 µg/mL), and supernatants were subsequently collected and measured by an enzyme-linked immunosorbent assay (ELISA) kit #ab201734 (Abcam, Cambridge, MA, USA).

### 2.8. Cytokine Analysis

As previously described [36], cytokine analysis was performed by the FirePlex Firefly^®^ and Analysis Workbench (Abcam, Cambridge, MA, USA), a software for multiplex protein expression assays, whereby supernatants were measured under LPS-induced inflammatory conditions, as described in Section 2.3, and compared with supernatants after subsequent addition of either N or ND treatments (63.34 μg/mL) during the last hour of the induction time. The cytokines investigated in both ARPE-19 and HREC cells included interleukin (IL)-1β, IL-6, IL -8, IL-10, IL-12p70, IL-18, interferon (IFN)-γ, monocyte chemoattractant protein (MCP)-1, and tumor necrosis factor (TNF)-α. All cytokines are expressed in pg/mL, except for MCP-1 and IL-8, which were expressed as ng/mL.

### 2.9. Statistical Analysis

To determine whether variables followed a normal distribution, we applied the Shapiro–Wilk and Levene tests to assess variance homogeneity. All parameters were subjected to analysis of variance (ANOVA) or Kruskal–Wallis test followed by Bonferroni post hoc test. A difference of *p* < 0.05 was considered statistically significant. GraphPad Prism 6.0 (GraphPad Prism Software Inc., San Diego, CA, USA) was used for statistical analysis.

## 3. Results

The phenotype of both ARPE-19 and HREC cells was stable under immunofluorescence staining with the corresponding RPE65 and caveolin markers (Figure S1), and no cytotoxicity was observed even at five times higher the optimized concentration by MTT assay (Figure S2).

### 3.1. Effect of N and ND Treatments on Junctional Integrity

**Figure 1:**
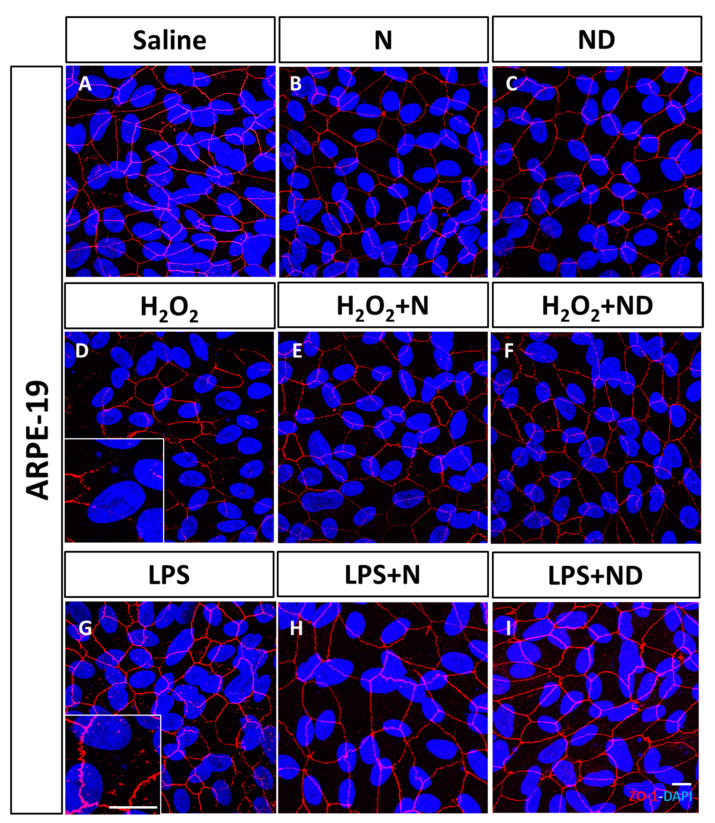
Junctional integrity of ARPE-19 cells evaluated by ZO-1 (red) fluorescence under a confocal imaging system. Application of N and ND treatments (62.34 μg/mL each) did not affect tight junctions, cell integrity, and structure (**B**,**C**) compared to saline (**A**). Addition of H_2_O_2_ (2h; 1600 μM) and LPS (24 h; 20 μg/mL) to induce oxidative stress and inflammatory-like conditions, respectively, damaged tight junctions (**D**,**G**), while incubation with N and ND treatments during the last hour of the induction recovered the altered structure (**E**,**F**,**H**,**I**). Nuclei were labelled with DAPI (blue). Scale bar: 20 µm.

In order to assess the effect of oxidative stress and inflammation on tight junctions of ARPE-19 cells, we examined ZO-1 by immunofluorescence. There were disruptions and a reduction of immunofluorescence staining in the integrity of the apical areas of epithelial monolayer under oxidative stress induced by H_2_O_2_ (Figure 1D) compared to saline conditions (Figure 1A). Similarly, ZO-1 immunofluorescent staining under LPS-induced inflammation showed a disruption in cell contact (Figure 1G) in comparison to basal conditions (Figure 1A). Application of either N or ND treatments to the cell line showed formation of stable tight junctions (Figure 1B,C) similar to saline conditions (Figure 1A). Interestingly, when N and ND treatments were added in H_2_O_2_- (Figure 1E,F) and LPS-induced media (Figure 1H,I), they recovered the effect of oxidative stress and inflammation (Figure 1D,G) contributing to maintenance of cell polarity, suggesting a protective effect of N and ND on tight junctions and integrity in ARPE-19 cells.

### 3.2. Effect of N and ND treatments on Apoptosis and Cell Proliferation

**Figure 2:**
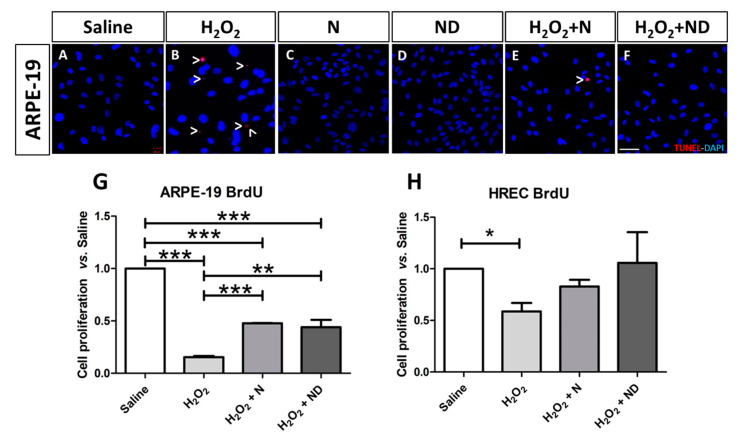
Late apoptosis assessed in ARPE-19 cells by TUNEL labelling and imaged under a confocal microscope. Application of N and ND treatments (62.34 μg/mL) to the media (**C**,**D**) showed similar results to saline (**A**). Addition of H_2_O_2_ (2 h; 600 μM) to induce oxidative stress increased TUNEL-positive-stained ARPE-19 cells (**B**; red), while TUNEL labelling was absent by application of N and ND treatments (62.34 μg/mL each) during the last hour of the induction (**E**,**F**). Nuclei were labelled with DAPI (blue). Scale bar: 50 µm. Cell proliferation assay was performed in both ARPE-19 and HREC cells (**G**,**H**), *n* = 3. BrdU expression levels were significantly reduced in both cell lines following oxidative stress-induced conditions by 1000 μM H_2_O_2_ for 2h. This effect was significantly recovered in ARPE-19 cells by application of N (*p* < 0.001) and ND (*p* < 0.01) treatments (62.34 μg/mL each) during the last hour of the induction, while a strong tendency towards significance was observed in HREC cells (* *p* < 0.05, ** *p* < 0.001, *** *p* < 0.001).

TDT-mediated dUTP-biotin nick end-labeling (TUNEL) revealed no presence of apoptotic ARPE-19 cells following addition of either N or ND treatments to the culture media (Figure 2C,D), similar to saline conditions (Figure 2A). Application of H_2_O_2_ to induce oxidative stress showed increased TUNEL-positive immunostaining of ARPE-19 cells (Figure 2B) compared to saline conditions (Figure 2A), which was counteracted by addition of either N or ND treatments to the oxidative stress-induced media (Figure 2C,D).

Oxidative stress plays a critical role in the development and progression of AMD. Increased reactive oxygen species may cause RPE cell dysfunction leading to AMD. Therefore, cell proliferation assay was performed in order to examine the antioxidant effect of the two formulations in recovering cell viability and to align the outcome with the apoptosis assay (TUNEL staining). In addition, cell proliferation, as assessed by BrdU expression levels, was significantly reduced in ARPE-19 (*p* < 0.001; Figure 2G) and HREC (*p* < 0.05; Figure 2H) cells subjected to H_2_O_2_-induced oxidative stress. After application of either N or ND treatment to the oxidative stress-induced media, there was significant recovery and increased cell proliferation compared to H_2_O_2_ alone (*p* < 0.001 and *p* < 0.01 for N and ND, respectively, Figure 2G) in ARPE-19 cells. In the case of HREC cells, there was a trend towards recovery of cell proliferation and increased BrdU levels, being more potent with addition of ND than N; however, it did not reach statistical significance (Figure 2H).

### 3.3. Antioxidant properties of N and ND treatments

**Figure 3:**
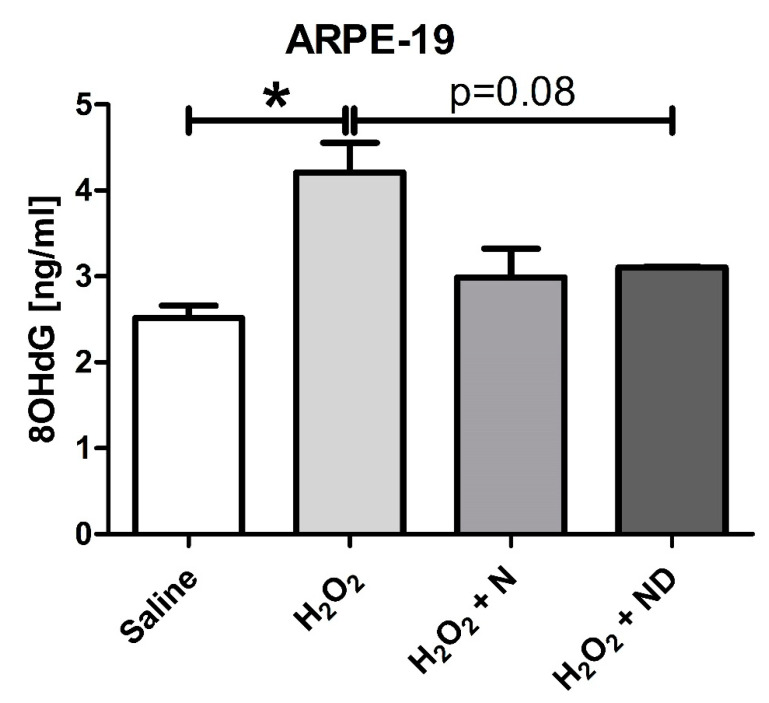
Oxidative damage to DNA was analyzed by 8OHdG expression levels for ARPE-19 cells measured by ELISA (*n* = 3). Addition of H_2_O_2_ (2 h; 1000 μM) to induce oxidative stress conditions significantly (*p* < 0.05) increased 8OHdG levels. Application of N and ND treatments (62.34 μg/mL each) during the last hour of the induction time were able to reduce 8OHdG levels in the media, but not to significant levels (*p* = 0.08). (* *p* < 0.05).

Figure 3 shows that oxidative stress induced by H_2_O_2_ significantly (*p* < 0.05) increased the expression levels of the marker representative for oxidative damage to DNA, 8-OHdG, in supernatants from ARPE-19 cells. Addition of either N or ND to the cells under induced oxidative stress resulted in a tendency to reduce 8-OHdG expression levels (*p* = 0.08 for ND).

### 3.4. Regulation of Inflammatory Cytokines by N and ND Treatments

**Figure 4:**
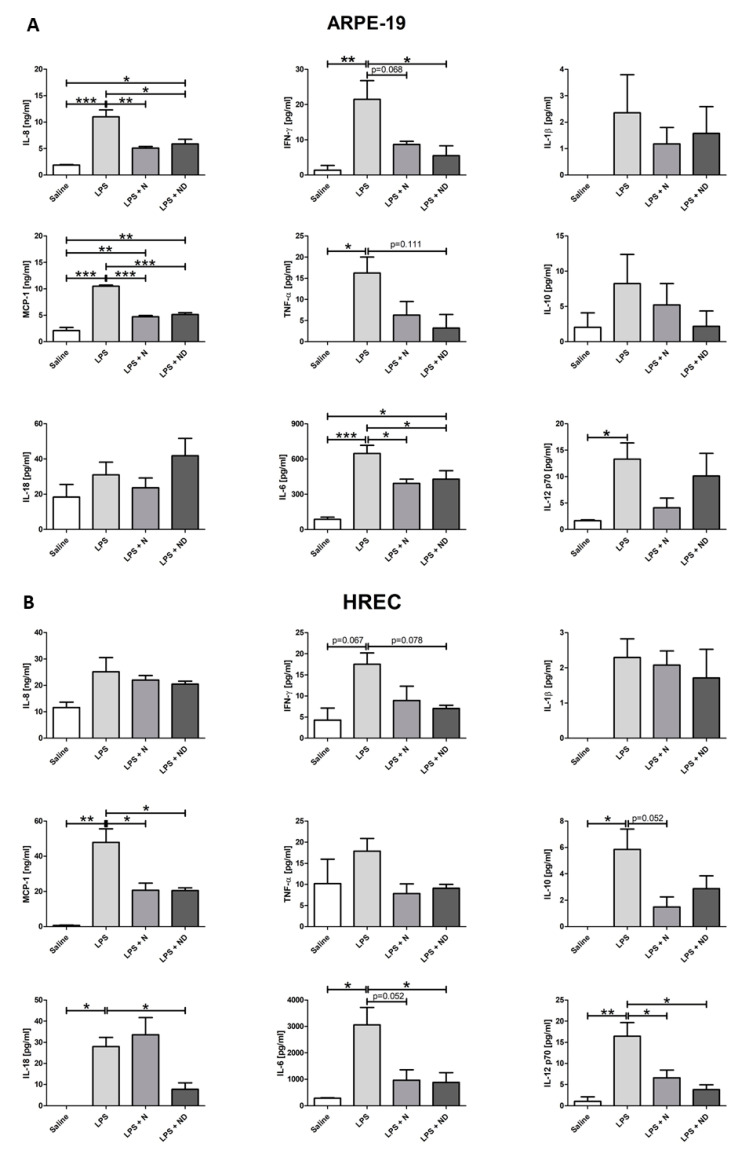
Multiple cytokine expression assays in ARPE-19 and HREC cells (*n* = 3). (**A**) Expression levels of IL-8, MCP-1, TNF-α, IFNγ, IL-6, and IL-12p70 were significantly increased (*p* < 0.05) following addition of LPS (24 h; 20 μg/mL) to induce inflammatory-like conditions in ARPE cells. IL-8, MCP-1, and IL-6 expression levels were significantly reduced (*p* < 0.05) following application of N and ND (62.34 μg/mL each) during the last hour of the induction time. Addition of ND in LPS-induced media was able to significantly decrease (*p* < 0.05) the expression levels of IFNγ and attenuate the expression of TNFα (*p* = 0.111), while N showed a strong tendency to reduce IFN-γ (*p* = 0.068). (**B**) Expression levels of MCP-1, IL-10, IL-18, IL-6, and IL-12p70 were significantly increased (*p* < 0.05) after addition of LPS to induce inflammatory-like conditions (24h; 50 μg/mL) in HREC cells. Expression levels of MCP-1 and IL-12p70 were significantly reduced (*p* < 0.05) following application of N and ND (62.34 μg/mL each) during the last hour of the induction time. Addition of ND, and not N, in LPS-induced media significantly decreased IL-6 and IL-18 expression levels (*p* < 0.05), while there was a tendency to downregulate IFNγ (*p* = 0.078). Addition of N attenuated expression of IL-6 and IL-10 (*p* = 0.052). All cytokines are expressed in pg/mL except for MCP-1 and IL-8 that were expressed as ng/mL. (* *p* < 0.05, ** *p* < 0.001, *** *p* < 0.001).

Figure 4A shows that inflammation induced by LPS significantly (*p* < 0.05) increased the expression levels of IL-8, MCP-1, TNFα, IFNγ, IL-6, and IL-12p70 cytokines in ARPE-19 cells. Under LPS-induced inflammatory-like conditions, application of either N or ND treatment to the media significantly (*p* < 0.05) downregulated the expression of IL-8, IL-6, and MCP-1. Significant reduction of IFNγ expression levels was observed only upon addition of ND (*p* < 0.05) and not N (*p* = 0.068). Interestingly, addition of N showed a strong tendency to reduce TNFα expression levels (*p* = 0.111) when added in inflammatory-induced ARPE-19 cells.

HREC cells subjected to LPS-induced inflammatory-like conditions showed a significant (*p* < 0.05) increase in the expression levels of MCP-1, IL-10, IL-18, IL-6, and IL-12p70 cytokines. Addition of either N or ND to LPS-induced media significantly (*p* < 0.05) attenuated expression levels of MCP-1 and IL-12p70. The expression levels of IL-6 and IL-18 were significantly reduced (*p* < 0.05) only after addition of ND and not N in LPS-induced cells. IFNγ expression levels revealed a tendency to increase (*p* = 0.067) after LPS addition, while application of ND treatment showed a tendency to downregulate the cytokine (*p* = 0.078). Interestingly, addition of N treatment to inflammatory-induced HREC cells had a strong tendency to decrease the expression levels of IL-10 and IL-6 (*p* = 0.052).

In our experiment, we noticed that the expression levels of few cytokines, in both ARPE-19 and HREC cells, under LPS-induced inflammatory conditions were not increased, and there was no significant difference with saline. A plausible reason could be the dispersion of controls in our study. In addition, absence of regulation of those proteins by LPS at the studied concentration could provide information on the expression pathway for each cell type.

## 4. Discussion

This study demonstrates that the oxidative and inflammatory-induced damage was reversed in ARPE-19 and HREC cell lines by adding either a nutritional antioxidant complex (N) or the complex in combination with vitamin D (ND). In some circumstances, ND appeared to have had a more potent effect than N alone, suggesting a possible synergistic role between the supplement and vitamin D.

Specifically, ARPE-19 cells, in response to oxidative stress or inflammation, induced by the addition of H_2_O_2_ or LPS, revealed partial loss and damage of their junctional integrity. This is in line with previous studies whereby exposure of ARPE-19 cells to the same stress inducers resulted in reduced expression of ZO-1 protein, more disorganized tight junctions with breaks in peripheral staining [36,44,45]. Additionally, our results demonstrated that application of N and ND to the media counteracted these morphological changes. Earlier investigations demonstrated that vitamin D restored damaged tight junctions and increased ZO-1 protein levels in ARPE-19 cells under oxidative stress conditions [36,46], which suggests its role in blood–retinal barrier integrity through intercellular adherent junctions [47].

8-OHdG is a major product of oxidative DNA damage [48] that is associated with AMD [49,50,51] and was used in our study as an additional oxidative stress marker to further confirm the antioxidant effect of vitamin D and the nutritional complex. A recent study has described a negative correlation between an aging suppressor protein in mammals, klotho, with 8-OHdG expression levels in aqueous humor of patients with exudative AMD [51]. This suggests that the reduced antioxidant defense response by the attenuated expression levels of klotho result in enhanced oxidative stress and consequently to AMD pathogenesis. The elevated 8-OHdG levels observed in ARPE-19 cells after H_2_O_2_-induced oxidative stress were reduced following addition of either N or ND to the media. Deficiency of vitamin D has been described to be associated with higher oxidation-induced DNA damage in diabetic retinopathy [52], a retinal disorder associated with diabetes mellitus [53], and supplementation with vitamin D had a beneficial impact on oxidative stress by reducing 8-OHdG concentrations in patients with type 2 diabetes [54]. Investigators have also demonstrated that elevated 8-OHdG levels in ARPE-19 cells under H_2_O_2_ oxidative stress-induced conditions were significantly reduced by application of vitamin D to the media [36]. Therefore, vitamin D may participate in antioxidant-related mechanisms to decrease oxidative stress by scavenging reactive oxygen species whose production in the retina result in mitochondrial and nuclear DNA damage as described for RPE in AMD patients [50]. In addition, the role of vitamin D in oxidative stress reduction was described in mouse cone cells against photoreceptor degeneration [55] and in studies involving upregulated expression of antioxidant genes (glutathione peroxidase GPX2 and GPX3; superoxide dismutase SOD1 and SOD2; catalase, CAT) [35].

Reactive oxygen species production can activate apoptosis signaling pathways [56], and antioxidants are able to suppress apoptosis [57]. Earlier studies have shown that H_2_O_2_, LPS, or high-glucose-induced oxidative damage enhance apoptosis in RPE and retinal endothelial cells [35,58,59,60,61], which was reduced following application of vitamin D in ARPE-19 cells [35,36,60] and retinal endothelial cells [36,61], respectively. This is consistent with our results, where we observed that H_2_O_2_ resulted in enhanced apoptosis in ARPE-19 cells, which was counteracted by the addition of either N or ND treatments to the media. In addition, our results demonstrate significant reduction in cell proliferation after addition of H_2_O_2_ in both cell lines, characterized by subsequent recovery following application of either N or ND to the media, with ND presenting a more potent effect than N in HREC cells. Earlier studies have effectively described the vital role of oxidative stress by H_2_O_2_ in inhibiting cell proliferation in various cells [62], including RPE in the retina [35,46,63,64]. The exact mechanisms have not been identified, however, recent studies have revealed the interference of H_2_O_2_ in numerous intracellular signaling pathways in retinal epithelial cells, such as the epidermal growth factor receptor/AKT and Notch signaling pathways [46,64], including the regulation of autophagy [65], kinases, and pro-apoptotic proteins such as caspases [66,67,68]. These studies open the way towards future identification of the mechanisms involved in the interplay between vitamin D and retinal cells in restoring cell proliferation and protecting RPE from angiogenesis.

Overall, the results above suggest that vitamin D possibly provides an additional protective effect to the nutritional antioxidant complex in RPE cell viability and the blood–retina barrier from chronic oxidative stress and apoptosis, which lead to retinal pathologies, including AMD.

In the present study, besides oxidative stress, we investigated the potential role of vitamin D and the nutritional antioxidant complex in the downregulation of inflammatory cytokines under LPS-induced inflammatory conditions.

Inflammatory mediators, such as IL-1β, IL-6, IL-8, IL-10, IL-18, IL-12p70, TNFα, IFNγ, and MCP-1, play a vital role in the development of AMD and have angiogenic properties [69,70,71,72,73,74,75,76,77,78], and their activity is upregulated following addition of LPS [36] or H_2_O_2_ [35] to the media.

Consistent with our observations, earlier studies have reported downregulation of IL-8, IL-6, MCP1, IFNγ, and TNFα in ARPE-19 cells [35,36,79,80] and reduced expression levels of MCP-1, IL-12p70, IL-18, IL-6, and IFNγ in HREC cells [36] following vitamin D administration to the media. Our results indicate that ND treatment more effectively downregulates the expression levels of IL-18, IL-6, and IFNγ in HREC cells and of IFNγ and TNFα in ARPE-19 cells under LPS-induced inflammation. Therefore, vitamin D potentially has a synergistic effect with the nutritional antioxidant complex in regulating cytokine expression levels. This further suggests future elucidation of understanding the critical molecular interactions between vitamin D and nutritional complex components in combination with the corresponding cytokines and intracellular signaling transduction pathways involved in potentially preventing progression to neovascular AMD.

The observed downregulation of IL-18 and IL-12p70 upon addition of vitamin D under LPS-induced inflammatory conditions only in HREC cells and not in RPE cells suggests an anti-angiogenic role through regulation of vascular endothelial growth factor (VEGF) production. This is consistent with an earlier study where IL-18–neutralizing antibodies were intravitreally injected in a laser-induced choroidal neovascularization (CNV) mouse model, and CNV development increased compared to non-injected mice [81]. However, the anti-angiogenic effect of IL-18 is not clear, and further investigation is required since other studies show that intravitreal injections of IL-18 in CNV mouse, and transfection of plasmids encoding IL-18 into RPE cells via injection in mice, had no effect on CNV volume [82].

The pro-inflammatory cytokine IL-12 enhances production of IFNγ through activation of the JAK/STAT signaling pathway [83], which along with TNFα and IL-1β lead to enhanced production of VEGF and IL-6 [77], whereby all of these mediators are implicated in phagocytosis, visual cycle, and epithelial morphology in RPE cells [70], as well as in the complement pathway, angiogenesis, and matrix remodeling [69]. Therefore, the observed downregulation of IL-8, IL-18, IL-12p70, and MCP-1 by vitamin D may potentially reduce the concentration of macrophages, microglia, and lymphocytes in the retina and inhibit migration and proliferation of endothelial cells that play a key role in angiogenesis [77,84].

Vitamin D has been shown to stimulate the expression of the potent anti-inflammatory cytokine IL-10 [85,86], which inhibits the production of the proinflammatory IL-12 through nuclear factor κB (NfκB) and activator protein 1 (AP1) [83], and of certain other proinflammatory cytokines, such as IL-1, IL-6, IL-8, and TNFα [32,83,86,87]. The anti-inflammatory role of IL-10 has been shown in a CNV mouse model, where IL-10 deficiency increased inflammation and subsequent intraocular injection enhanced the volume of neovascularization [88]. Similarly, IL-10 increased the secretion of the proinflammatory cytokine IL-6 through regulation of VEGF production and is implicated in the prevention of the development of rhematogeneous retinal detachment [89]. Interestingly, our results are consistent with a recent study [36], revealing that the elevated IL-10 expression levels under LPS-induced inflammatory conditions remained unmodified after application of either N or ND in both cell lines, thus suggesting a protective role of vitamin D against inflammation. Additionally, the IL-10 downregulation, after addition of ND under inflammatory-induced conditions, was higher in ARPE-19 than HREC cells, supporting a potential anti-angiogenic role through a synergistic effect between the nutritional antioxidant complex and vitamin D by inducing IL-10, which in turn is involved in a dynamic modulation of pro-inflammatory cytokines and transcription factors.

This interplay of molecular interactions and involvement of various signaling pathways requires deeper investigation while considering the involvement of other cytokine mediators not mentioned in this study. For instance, vitamin D has been recently shown to recover expression levels of IL-33 in ARPE-19 cells after H_2_O_2_ addition, which stimulated expression of antioxidant genes [35], suggesting a key role of this cytokine in AMD since it was also shown to inhibit murine CNV [90] and to reduce retinal inflammation [91].

One limitation of this study involves the experimentation on immortalized cell lines, and thus we recommend future investigation on primary cell lines and subsequently in in vivo models of AMD and clinical trials. This will enable us to take into consideration other molecules, such as natural nutritional components, vitamins, and minerals, that may also provide an additional benefit in combination with vitamin D.

Additional future studies could examine modulation of matrix metalloproteinases (MMPs) and VEGF/PEDF by N and ND under oxidative stress-induced conditions. MMP activity in RPE affects the ECM and subsequently the thickness of Bruch membrane, which may induce release/activation of angiogenic factors that are vital to angiogenesis, such as VEGF/PEDF, from choroidal endothelial cells. Therefore, endothelial cells in the choroid could be investigated since different cell types may respond differently to N and/or ND under oxidative or inflammatory stress-induced conditions and may involve different signal transduction mechanisms. This will further enable us to evaluate the signaling events among the cells in the RPE, the retina, and the choroid, and thus further elucidate retinal homeostasis.

## 5. Conclusions

Our results demonstrate that upon oxidative stress and inflammation-induced conditions, cell proliferation is reduced, junctional integrity is weakened, oxidative DNA damage and apoptosis are enhanced, and expression levels of inflammatory cytokines are increased. Nevertheless, application of vitamin D together with the nutritional antioxidant complex to the media counteract these changes with ND, exhibiting in some cases a more potent protective role than N alone, suggesting the possible existence of a synergistic effect between the nutritional antioxidant complex and vitamin D towards a more efficient oral supplementation treatment for the early stage of AMD.

## Data Availability

All data are available within the manuscript and upon request to corresponding authors.

## Supplementary Materials

The following are available online at https://www.mdpi.com/2072-6643/13/5/1423/s1, Figure S1: ARPE-19 and HREC cells’ phenotyping. Figure S2: MTT assay in ARPE-19 and HREC cells. Table S1: Composition of Nutrof Total^®^ used in the study (per one capsule).
