# Supplementary material for: Anti-Inflammatory and Anti-Oxidative Synergistic Effect of Vitamin D and Nutritional Complex on Retinal Pigment Epithelial and Endothelial Cell Lines against Age-Related Macular Degeneration
Source: Nutrients. 2021 Apr 23;13(5):1423. doi: 10.3390/nu13051423 (PMC8170899; doi:10.3390/nu13051423)
Supplement: Supplementary file 1 [file nutrients-13-01423-s001.zip › Supplementary figures and table.docx]

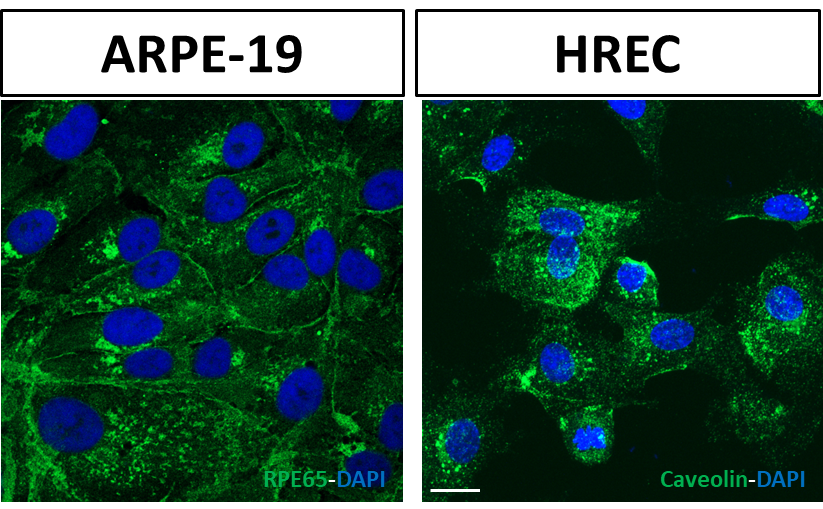


Figure S1. ARPE-19 and HREC cells’ phenotyping. Immunofluorescence labelling of RPE65 (A; green) and caveolin (B; green) for ARPE-19 and HREC cell lines’, respectively, imaged under confocal microscope. Nuclei were labeled with DAPI (blue). Scale bar: 20 µm.


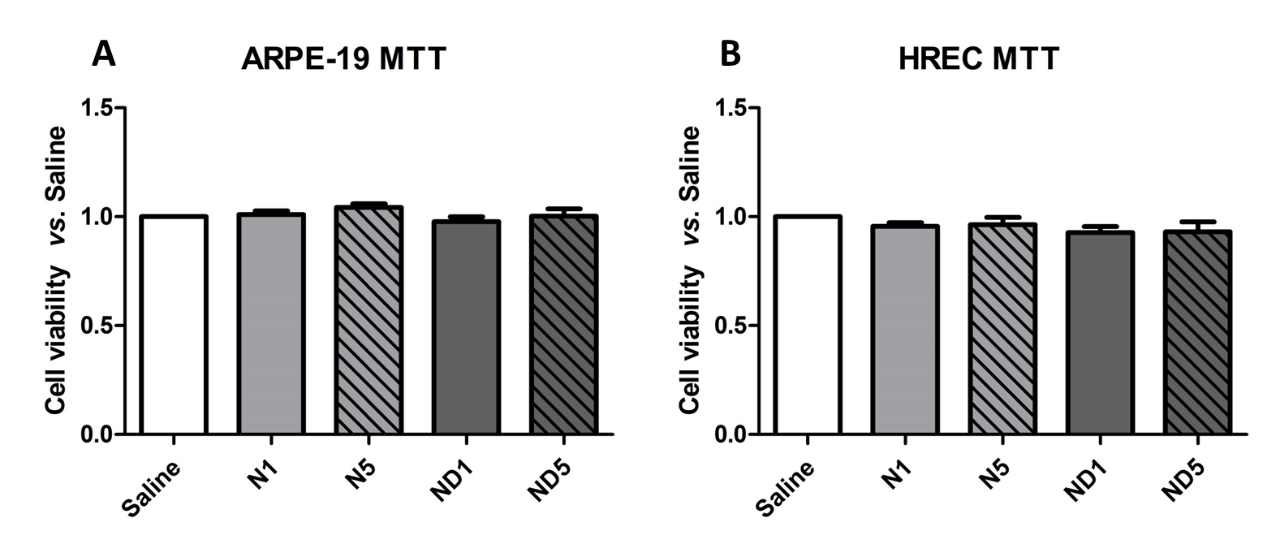


Figure S2. MTT assay in ARPE-19 and HREC cells. MTT assay showed no cytotoxicity in (A) ARPE-19 and (B) HREC cells after exposure for 24h to either N (1nM) or ND (62.34 μg/ml) treatments at both tested concentrations (N5/ND5 denotes five times more the concentration of N/ND, respectively, i.e. ND1=5nM, ND5=311.7μg/ml). Abbreviations: N: nutritional antioxidant complex, ND: nutritional antioxidant complex + vitamin D.

Table S1**.** Composition of Nutrof Total® used in the study (per one capsule).

|  | **Nutrof Total**® **(mg)** | **Nutrof Total**® **+ vitaminD (mg)** |
| --- | --- | --- |
| Vitamin C | 60 | 60 |
| Vitamin E | 10 | 10 |
| Vitamin D3 | - | 0.005 |
| Zinc | 10 | 10 |
| Selenium | 0.025 | 0.025 |
| Copper | 0.5 | 0.5 |
| Lutein | 10 | 10 |
| Zeaxanthin | 2 | 2 |
| Fish oil | 330 | 330 |
| *with* | *132 EPA 66 DHA* | *132 EPA 66 DHA* |
| Glutathione | 1 | 1 |
| Resveratrol | 1 | 1 |
